# Re-Examining the Importance of Pigs in the Transmission of Japanese Encephalitis Virus

**DOI:** 10.3390/pathogens11050575

**Published:** 2022-05-13

**Authors:** So Lee Park, Yan-Jang S. Huang, Dana L. Vanlandingham

**Affiliations:** 1 Department of Diagnostic Medicine and Pathobiology, Kansas State University, Manhattan, KS 66506, USA; parksolee@vet.k-state.edu (S.L.P.); yshuang1985@ksu.edu (Y.-J.S.H.); 2 Biosecurity Research Institute, Kansas State University, Manhattan, KS 66506, USA

**Keywords:** Japanese encephalitis virus (JEV), pig, transmission, oronasal shedding, amplifying host

## Abstract

Japanese encephalitis virus (JEV), a mosquito-borne flavivirus, is the leading cause of pediatric encephalitis in Southeast Asia. The enzootic transmission of JEV involves two types of amplifying hosts, swine and avian species. The involvement of pigs in the transmission cycle makes JEV a unique pathogen because human Japanese encephalitis cases are frequently linked to the epizootic spillover from pigs, which can not only develop viremia to sustain transmission but also signs of neurotropic and reproductive disease. The existing knowledge of the epidemiology of JEV largely suggests that viremic pigs are a source of infectious viruses for competent mosquito species, especially *Culex tritaeniorhynchus* in the endemic regions. However, several recently published studies that applied molecular detection techniques to the characterization of JEV pathogenesis in pigs described the shedding of JEV through multiple routes and persistent infection, both of which have not been reported in the past. These findings warrant a re-examination of the role that pigs are playing in the transmission and maintenance of JEV. In this review, we summarize discoveries on the shedding of JEV during the course of infection and analyze the available published evidence to discuss the possible role of the vector-free JEV transmission route among pigs in viral maintenance.

## 1. Introduction

Japanese encephalitis virus (JEV) is an enveloped positive-sensed RNA virus belonging to the *Flavivirus* genus in the *Flaviviridae* family [1]. Most pathogenic flaviviruses, for example, JEV, are arthropod-borne viruses that are maintained through the biological transmission between viremic vertebrate hosts by hematophagous arthropods such as mosquitoes [2,3,4]. In other words, arboviruses require susceptible vertebrate hosts that can produce sufficient viremia that is capable of infecting biting competent arthropods for them to pass on the virus to new vertebrate hosts in the same transmission cycle [4]. For JEV, pigs and birds play this important role of amplifying hosts, which helps sustain the enzootic transmission cycle in the endemic regions [5]. There are many other different vertebrate host species that are susceptible to JEV infection, including cattle, goats, horses, dogs, bats, reptiles, chickens, and humans [6,7,8,9]. However, most of them are considered incidental or dead-end hosts because they are not capable of developing sufficient viremia required for transmission.

While the biological transmission by hematophagous arthropods is the primary route of infection and disease of arboviruses, flaviviruses have historically been proven to be capable of being transmitted without the simultaneous presence of viremic hosts and competent vectors. The advancement in molecular techniques in the detection of viral RNA, several laboratory transmission studies, and some case reports have hinted at the possibility of vector-free transmission of flaviviruses in nature, which includes the direct exposure, primarily of mucosal surfaces, to infectious viruses shed by the infected vertebrate host. Examples of these non-vector-borne transmission routes include the oral transmission of tick-borne encephalitis virus from the consumption of raw milk or cheese produced from infected animals [10,11,12], the route of sexual transmission of Zika virus [13,14,15], and the direct contact transmission of West Nile viruses (WNV) between birds possibly via oral and cloacal shedding [16].

In the last decade, growing evidence has been accumulating that suggest transmission of Japanese encephalitis (JE) can also be facilitated through the oronasal shedding between pigs [17,18,19], highlighting the possibility that the enzootic transmission cycle of JEV may be more complicated that initially perceived. This review will discuss the significance of this unconventional route of transmission in the maintenance of JEV in nature and how it could potentially reframe the importance of pigs in JEV transmission.

## 2. Epidemiology and Ecology of JEV

Japanese encephalitis virus is an encephalitic flavivirus that is capable of causing fatal infections of the central nervous system in immunologically naïve humans [20]. It is currently endemic to the Asian-Pacific region. With no specific treatments available, the prevention of JE via vaccination is paramount [20]. There are currently several inactivated and live attenuated licensed vaccines available as intramuscular or subcutaneous doses that can provide effective means of protection from infection and subsequent disease [21,22]. The three types of licensed vaccines available at this time are inactivated Vero cell-derived vaccines (based on Beijing-1 or SA14-14-2 strains), live attenuated vaccine (JEV SA14-14-2), and live chimeric vaccine (ChimeriVax-JE) [21,22,23]. While vaccination is the most effective tool for the control of JEV, approximately 68,000 cases are still reported each year in endemic countries, of which about 75% occur in children under 15 years of age [20,24,25]. Clinical symptoms in infected humans can range from nonspecific flu-like symptoms, including headache, high fever, and lethargy, to severe clinical manifestations such as paralysis, motor and memory deficits, and seizures [7,26]. To date, there have been five distinct genotypes of JEV identified based on sequences of the flaviviral envelope protein [27]. The majority of human diseases are caused by JEV strains belonging to the clade b of genotype I (GI-b) and genotype III (GIII) [28,29]. Importantly, the emergence of JEV GI-b has been reported in multiple regions, leading to the displacement of the previously endemic GIII strains [29].

The transmission of JEV by competent mosquito species fulfills the criteria for the biological transmission of arboviruses, which is the multiplication of JEV in both mosquitoes and amplifying hosts. To date, there have been more than 10 mosquito species proven to be competent for the transmission of JEV [30,31]. Amongst these mosquitoes, *Culex* species mosquitoes are the predominant competent vectors for JEV due to their zoophilic feeding pattern [32]. By definition, competent vector species must be capable of acquiring the virus infection in nature, transmit the infection by feeding on susceptible vertebrate hosts, and be abundant enough to be significant [33]. The major vectors that fit these criteria include *Culex tritaeniorhynchus*, *Cx. gelidus*, *Cx. vishnui*, and *Cx. annulirostris* [5,33]. From this short list, *Cx. tritaeniorhynchus* is recognized as the principal vector of JEV due to its high susceptibility, transmission rate, and wide distribution [33,34,35]. The significance of these major mosquito vectors may evolve with time as climate change, including global warming and increased flooding, continues to impact vector abundance and geographic distribution [5,36,37].

While *Culex* species mosquitoes function as major vectors, viremic swine and avian hosts, particularly the water-wading birds of the *Ardeidae* family, are the major sources of infectious viruses [5,33,38]. Japanese encephalitis is primarily considered a rural agricultural disease, with epizootic spillover of JEV driven by the close association between the humans and amplifying hosts [32]. For example, the intense farming of domestic pigs, especially those of backyard farming, is attributed to the increased risk of human JEV infection in the endemic regions [5,32]. The rapid birth rate and turnover of pigs in these farms constantly generate susceptible populations that can help amplify JEV and maintain its presence in the region [32,39]. Additionally, the predilection of *Cx. tritaeniorhynchus* to feed on pigs further support the role of pigs as important amplifying hosts [40,41]. As such, the ecology of JEV is unique among encephalitic flaviviruses because of the involvement of domestic farm animals. Urbanization and increased agricultural productivity continue to move pigs and rice farming closer to peri-urban and/or urban areas, increasing the concern of their impact on host abundance and their availability to vectors [5,36].

Although the major players of JEV transmission have been identified, there are several knowledge gaps in the transmission and maintenance of JEV in its enzootic cycle, because only a limited number of studies have characterized the course of JEV infection in amplifying hosts, especially domestic pigs. Most of our understanding of JEV pathogenesis in domestic pigs thus far have been based on the detection of infectious viruses or viral genome in the serum and brain of naturally infected animals [42,43,44,45].

## 3. JE Disease in Swine

It is well accepted that pigs develop viremia to sustain the enzootic transmission and facilitate the epizootic spillover of JEV. Japanese encephalitis virus infection and subsequent disease in pigs are generally mild and age-specific [7]. Neurotropic disease is more likely to be observed in young pigs. However, natural infection and disease in young piglets are not commonly reported from the endemic areas, possibly due to the presence of maternal antibodies, which can last up to six months of age [46,47]. While non-specific clinical signs such as fever, anorexia, and depression are observed early with JEV infection, neurologic signs such as hind limb tremors or ataxia can sometimes develop after five days post-infection [48,49,50,51,52]. Some infected pigs can progress into developing a wasting-like syndrome [52,53].

Infection of JEV in sexually matured adult pigs can result in reproductive failure in the form of abortions and transient infertility, resulting in significant implications for the pork and swine industry [42,54,55,56,57]. Abortions, abnormal farrowing, mummified fetuses, and weak piglets are most commonly observed if the pregnant sow became infected before 60 to 70 days of gestation [46,56]. Reports estimate that approximately 40% to 53% of unvaccinated pregnant sows had stillbirths and abortions in Japan during the epidemic seasons between 1947 and 1969 [54,58,59]. Reproductive disease from JEV infection can also affect boars. Infected boars can develop edematous or congested orchitis with abnormal spermatozoa but are capable of recovering completely most of the time [60,61].

Despite the potential for reproductive disease and JEV-infected pigs as a source for epizootic spillover, there are currently no licensed JEV vaccines for pigs. There are only regionally approved vaccines, including live attenuated at222, ML17, and anyang300 vaccines, that are available for local use in Japan, China, and Korea [27,48,62,63]. While reduction in JE disease and viremia in swine may be possible through pig immunization, it is important to understand that vaccination of domestic pigs cannot be solely relied upon to prevent the risk of human JEV infection and disease to the same extent as direct human immunization [64]. In addition to the rapid turnover of the pig population, the associated high costs, and the logistics of implementing a new swine vaccination program, alternate vertebrate hosts, such as wild feral pigs or birds, can still amplify the virus and maintain the transmission cycle in the area [64,65]. As such, swine immunization may be helpful to reduce disease in the pig population, but its contribution to reducing transmission risk to humans may not be significant and is probably relatively minimal.

The investigation of JEV pathogenesis in pigs has focused on the kinetics of viremia, which is directly relevant to the transmission of JEV, and the characterization of neurotropic disease, which resembles human JE. As the sensitivity of molecular detection techniques increased, several studies have detected JEV in various types of samples, including the oronasal shedding during the acute phase of infection and in lymphoid and nervous tissues during the convalescent phase of infection. While viremia in pigs remains central to the enzootic transmission of JEV as a mosquito-borne flavivirus, there may be other pathological outcomes that are relevant to the transmission of JEV but have not yet been examined in detail.

## 4. JEV Viremia in Infected Pigs: An Important Pathological Outcome Propelling the Biological Transmission of JEV

To date, laboratory studies have demonstrated that domestic pigs from various geographic regions are all susceptible to JEV and can be infected with the representative strains of endemic GI-b and GIII using different routes of infection, including intravenous, subcutaneous, intradermal, and intranasal challenge [19,51,52,53,66]. Similar pathologic outcomes and immune responses were observed in these infected pigs regardless of the challenge modality [19,66]. Several of these challenge studies and their major findings are summarized in Table 1.

**Figure 1:**
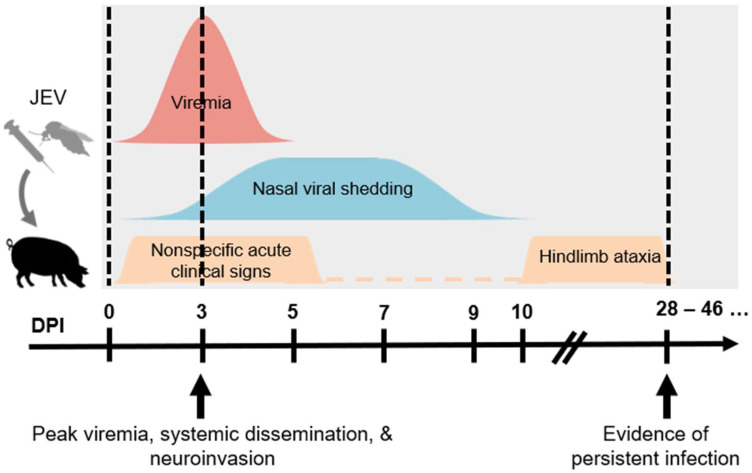
Schematic diagram summarizing the infection outcomes of JEV in pigs. Young piglets infected via different parenteral routes can result in similar pathologic outcomes including viremia, nasal viral shedding, clinical signs, and viral persistence. (JEV = Japanese encephalitis virus. DPI = day post-infection.).

The course of JEV infection in pigs involves the development of viremia, systemic infection, neuroinvasion, and persistent infection, as summarized in Figure 1. Viremia can be detected as early as 1-day post-infection (dpi) and persists for 4 to 5 days, and it is somewhat surprising that there are no demonstrable differences in the level and kinetics of viremia between different routes of challenge [19,66]. The highest viremic titers generally exceed 10^5^ 50% tissue culture infectious dose (TCID_50_)/mL or 10^6^ plaque forming unit (PFU)/mL between 1 and 5 dpi [19,50,51,66,68]. These viremic titers have been proven to be sufficient for the infection of *Cx. tritaeniorhynchus* [69]. Published studies have proven that greater than 50% of *Cx. tritaeniorhynchus* can be infected through the *per os* route using blood meals with infectivities of approximately 10^5^ and 10^6^ PFU/mL [69,70]. These observations confirm the role of domestic pigs in supporting the biological transmission of JEV.

Since the magnitude and duration of viremia in an amplifying host could potentially affect the prevalence and distribution of certain viral strains or genotypes, it is important to investigate the infection outcomes of different JEV genotypes in pigs. Importantly, the comparison of the ability of the two endemic JEV genotypes to induce viremia in pigs can be undertaken to investigate whether or not the displacement of GIII by the dominantly circulating GI-b is due to the increased ability of JEV GI-b to replicate to high titers in pigs and consequently facilitate the enzootic transmission. This hypothesis was directly tested by the comparison of viremic titers between the GIII CH1392 strain and the GI-b YL2009-4 strain in 10-week-old pigs [71]. The YL2009-4 strain (approximately 5 log_10_ focus forming unit (FFU)/mL) was reported to multiply to higher titers than the CH1392 strain (approximately 3 log_10_FFU/mL) at least at 2 dpi. Other experiments have also proven that another GI-b strain, JE-91, can also multiply to approximately 5 log_10_PFU/mL between 2 and 3 dpi [51]. These observations imply that JEV strains belonging to GI-b may have the fitness advantage over JEV GIII strains, and one cannot exclude that the higher viremic titers in pigs may facilitate the enzootic and epizootic transmission of GI-b JEV in specific ecological conditions. However, the results should be interpreted with caution. Firstly, there are some reports that have shown that the prototypic Nakayama strain (GIII) is capable of replicating to a comparable viremic titer [19,68]. In contrast to the study by Fan, et al. [71], another research group demonstrated that there was no difference between GIII and GI in the magnitude or duration of viremia in pigs [72]. Because the infectious dosage required for the infection of mosquito species competent for JEV is largely unknown, it is still unclear if the difference in the viremic titers between GI-b and GIII strains in pigs can be translated into the difference in the efficiency in the enzootic transmission. Additionally, as the genotype V (GV) of JEV has been reported to emerge in multiple countries in Asia [63,73,74], it may also be worthwhile determining the kinetics of viremia induced by GV to proactively investigate (1) whether or not the emergence of GV involves viremic pigs and (2) whether GV has any fitness advantage over the other two endemic genotypes, GI-b and GIII.

## 5. Systemic Spread and Neuroinvasive Phenotype of JEV

The development of viremia leads to the rapid and systemic spread of JEV in pigs. Viral dissemination and neuroinvasion coincide with the peak of viremia, leading to the detection of infectious viruses and viral genome in multiple types of tissues [50,51,68,75]. In contrast to other mammalian species, the neuroinvasive phenotype of JEV does not cause lethal diseases in pigs. While extensive pro-inflammatory cytokines are observed in the brain after JEV infection in humans, primates, and mice, JEV replication in the brain of pigs is mostly efficiently suppressed, predominately by type I interferon-independent activation of OAS1 (2′-5′-oligoadenylate synthetase 1) expression and increased interferon-gamma activity [66]. For example, the prototype Nakayama strain has a 50% lethal dose in ICR mice challenged via the intraperitoneal route at 0.5 PFU [76]. At the same time, the same strain does not produce lethal disease in pigs challenged via injection or oronasal routes [19]. Therefore, the experimental challenge of domestic pigs with the wild-type strains of JEV provides the unique opportunity to investigate the kinetics of neuroinvasive disease caused by JEV and the clearance of JEV from nervous tissues. This has been achieved in multiple published studies by monitoring infected pigs for several weeks and obtaining tissues samples through the course of infection [19,50,51,66,77]. These studies often combine the classical virology and contemporary molecular detection techniques to detect the presence of JEV genome in a variety of tissues. Surprisingly, the outcomes have not only delineated the tissue tropism of JEV in greater detail but have also revealed the persistent JEV infection even weeks after challenge [19,50,51,66,77]. In addition to the better understanding of the biology of JEV, these observations have significant implication for the chronic infection with encephalitic flaviviruses, which has only been examined in a limited number of animal models to date [78,79,80].

The neuroinvasive phenotype of JEV is not known to contribute to its transmission but has been observed in domestic pigs up to 9 weeks of age in experimental challenge [66]. Young pigs are prone to develop lesions in the central nervous system and signs of nonsuppurative encephalitis, consisting of perivascular cuffing with lymphocytes, multifocal gliosis, and neural degeneration and necrosis, are most prominent at 5 dpi [19,48,52,68]. The dissemination of JEV in lymphoid tissues is a hallmark of systemic infection but typically only demonstrate slight follicular hyperplasia [19].

Importantly, there is a growing number of reports showing that the RNA genome of JEV can be detected in various tissues of experimentally challenged animals even weeks after the pigs recover from the acute infection and develop neutralizing antibody responses. While JEV infections are primarily described as acute infection and disease, persistence of JEV RNA can be detected in the tonsil [19,50,51,77] and brain [19,51] of infected pigs almost a month after initial infection, suggesting that the virus may somehow be hidden from the host immune response. The investigation of persistent JEV infection to date has been limited to a few mouse models [80,81,82,83]. Therefore, the mechanism that leads to the persistence of viral RNA remains poorly understood. The epidemiological importance of persistent infection in the maintenance of JEV in nature remains to be ultimately confirmed with field studies. A critical but unanswered question is whether or not pigs can develop persistent JEV infection in nature and consequently develop viremia sufficient to support the biological transmission of JEV even months after the initial infection. Although rare, persistent JE infection and recrudescence of symptoms have been reported in human cases [32,84,85]. As the persistent infection has also been described in humans infected with WNV [86], a related encephalitic flavivirus, the investigation of persistent JEV infection in pigs may also shed light on (1) how encephalitic flaviviruses interact with the vertebrate hosts in the convalescent phase of infection and (2) how persistent infection of flaviviruses can play a role in viral maintenance.

## 6. Oronasal Shedding of JEV: Can Transmission Take Place Directly among Pigs?

The higher sensitivity of molecular detection techniques has revealed a more expansive list of tissues that can support the replication of JEV. Most significant is the detection of viral genome in oral and nasal shedding, first discovered by Ricklin, et al. [19] and subsequently confirmed by other independent studies via molecular detection and/or virus isolation [50,51,66,75,77,87]. Interestingly, the nasal shedding of live infectious viruses from infected pigs has been shown to facilitate the direct transmission of JEV to immunologically naïve pigs under laboratory conditions [19]. Ricklin, et al. [19] demonstrated that pigs are highly susceptible to oronasal infection with viral titers as low as 10 TCID_50_. Exposure via oronasal route, either via intranasal inoculation or direct nose-to-nose contact with infected pigs, led to viremia, systemic infection, and antibody production comparable to pigs infected via needle routes of challenge [19,66]. Although the serum viral loads were lower by 2 logs, there was no demonstrable difference in viral loads in various central nervous tissues [66]. Other animals have been demonstrated to be susceptible to JEV via oronasal and/or intranasal challenge, such as macaques, mice, and guinea pigs [88,89,90,91,92]. Mucosal transmission could also be theoretically possible in humans based on the recent evidence of oral shedding of viral RNA in JE patients detected via throat swab sampling [93]. However, pigs are unique in that they can themselves function as the source of virus after infection as viral shedders to potentially infect other pigs via direct transmission.

Despite subsequent investigations, the exact mechanism behind the oronasal shedding of JEV remains unclear. The shedding source is most likely a combination of virus released directly from the nasal epithelium or olfactory neuroepithelium [50,89] and an indirect reflection of blood as oral mucosal transudate [94], but not necessarily from the tonsils as previously suspected [77]. The hypothesis that the shedding source could be the central nervous tissue via the olfactory pathway may be supported by the detection of viral genome in different regions of brain days after the viremic level has fallen below the limit of detection [51,68].

This oronasal shedding of JEV has significant implication for veterinary diagnosis and virological surveillance. The collection of oral fluid and nasal secretions from infected pigs utilizes simple techniques. Lyons, et al. [87] have reported the method of collecting JEV-positive oral fluid samples using cotton ropes that are readily available. Therefore, it is anticipated that the integration of the same method into the existing veterinary diagnostic programs will be practical. Currently, the diagnosis of JEV in pigs can be based on virus isolation on central nervous system tissues; viral RNA detection in samples such as blood, brain, placental tissues, and cerebrospinal fluid; and/or via the detection of JEV-specific antibodies in cerebrospinal fluid or serum samples [20,95]. However, the collection of the samples needed to perform these World Organisation for Animal Health (OIE)-recommended diagnostic tests are often invasive, time-consuming, and require technical or veterinary expertise. Oronasal shedding of JEV can persist for almost two weeks based on viral genome detection and/or virus isolation [19,50,87], which is significantly longer than the duration of the viremic phase. Hence, it is not surprising that the detection of JEV in oronasal specimens is superior to the detection of JEV in swine serum, which is commonly used for virological surveillance. In a recently published field study by Chiou, et al. [96], the detection of the JEV genome via RT-PCR in swine oronasal specimens coincided with the detection of JEV in mosquitoes at the early phase of the epidemic season, providing the possibility of improving the virological surveillance programs in the endemic region. Interestingly, viral oronasal shedding was undetectable outside the mosquito season, suggesting that direct oronasal transmission may not play such a significant role in supporting overwintering of JEV in temperate regions [96].

**Figure 2:**
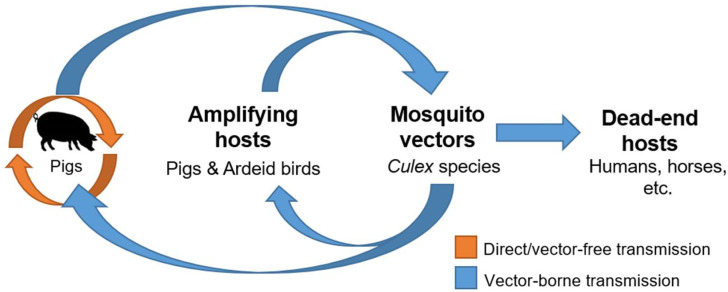
The proposed transmission cycle of JEV. Direct field data or studies are still lacking to support the existence and significance of vector-free transmission of JEV in nature. (JEV = Japanese encephalitis virus).

To date, the epidemiological importance of oronasal shedding of JEV from infected pigs remains to be proven. Available evidence that oronasal shedding facilitates JEV transmission among pigs is indirect (Figure 2). Serological data from other independent studies [17,18,97] support the potential existence and contribution of direct transmission in JEV ecology. Despite the very low to undetectable infection rates of JEV in field-collected mosquitoes, intense circulation of JEV was evident in pigs sampled in the same region based on the high seropositive prevalence or infection outbreaks reported in several studies [17,18,97], which could be a reflection of the existence of direct transmission of JEV between pigs. The existence of direct pig-to-pig transmission under field conditions has also been further supported using mathematical modelling consistent with swine serological data collected from Cambodia, a country with high JE incidence [18]. In this study, the mathematical JE transmission model that incorporated both vector-borne and direct transmission better fit the cross-sectional serological survey data collected from pig farms across different provinces of Cambodia compared to the model built on vector-borne transmission alone [18]. It is important to elucidate whether or not oronasal shedding will facilitate viral maintenance in the absence of mosquitoes, as demonstrated with laboratory studies. This may fulfill the missing knowledge of how JEV can be maintained in temperate regions of Asia, where competent mosquito species are not present year-round. To answer the question of whether or not this type of transmission is ecologically important requires multi-year data, preferably across multiple seasons, and virus genetic evidence, ideally through the detection of live viruses. The oronasal route of transmission could be important under different specific ecologic conditions, and identifying the major variables at play may be important to improve our knowledge of this unconventional transmission route.

## 7. Conclusions

Recent findings have demonstrated that JEV disease pathogenesis in pigs may be more complicated than initially perceived thanks to the detailed examination of tissue tropism and shedding profiles using molecular detection techniques [19,50,66,68]. Infected pigs can not only shed sufficient virus in their oronasal secretions that may be capable of infecting other pigs via direct transmission [19] but can also develop persistent infection of their lymphoid and/or nervous tissues [19,50,51]. Since its original discovery by Ricklin, et al. [19], the existence of direct vector-free transmission between pigs has been further in agreement with serosurveillance data from various pig farms [17,97] and with mathematical modelling [18]. This discovery of direct transmission is important as it raises a big question: Can flaviviruses be amplified and maintained in reservoir or amplifying hosts without the presence of competent vectors (Table 2)? This is a critical question of epidemiological importance because many zoonotic flaviviruses, including JEV, can be found in temperate regions in which vector-borne transmission is unlikely to take place year-round. If proven true, then an additional question must be investigated: How does this shape the evolution of JEV? The majority of available JEV isolates from pig samples are from blood, brain, and/or fetal materials, whereas there is a poor representation of viruses maintained through the oronasal process. Oronasal fluid sampling via the rope method is a type of technique proven to work in the field [96]. Therefore, more work can be conducted using this method to provide evidence that direct transmission of JEV occurs in nature and to determine its importance in the overall transmission and maintenance of the virus. If viruses are indeed transmitted under different conditions and transmission routes, it would be very interesting to investigate if there are any different genetic signatures that exist between the various populations.

## Figures and Tables

**Table 1:** Examples of JEV challenge studies using different challenge modalities.

Animals	Challenge Routes and Inocula	Major Finding(s)	Reference
10- to 20-day-old piglets of local breed from India (groups of *n* = 3–4)	SQ with 10^4^ to 10^5^ mouse LD_50_ JEV 897,795 strain; bite by JEV-infected *Culex vishnui* (genotype undetermined)	Pigs infected via SQ or infected mosquito bites developed similar magnitude and duration of viremia WNV infection provided partial cross-reactive immunity in pigs against JEV	[67]
3-week-old SPF piglets (groups of *n* = 1–3)	IV with ~10^6^ TCID_50_ JEV IB 2001 or AS-6 strains (genotype undetermined)	JEV-induced encephalitis in pigs was characterized Immunohistochemical distribution of viral antigens of JEV and the neurotropism of JEV were demonstrated in JEV-infected pigs	[53]
3-week-old SPF piglets (groups of *n* = 2)	IN with ~10^6^ TCID_50_ JEV IB 2001 (genotype undetermined)	IN challenge resulted in similar clinical signs, immunohistochemical distribution of JEV antigens, and histopathologic lesions as previously observed with IV challenge	[52]
7-week-old Swiss Large white pigs (groups of *n* = 2–3)	ID/IV with 10^6^ to 10^7^ TCID_50_ JEV Nakayama strain (GIII); Oronasal with 10^3^ to 10^7^ TCID_50_ Nakayama; ID or IV with 10^6^ TCID_50_ JEV Laos strain (GI)	Vector-free transmission of JEV was demonstrated experimentally in pigs Similar pathogenesis can be observed regardless of the different modes of infection and JEV genotype	[19]
9-week-old Belgian Landrace and Petrain cross pigs (groups of *n* = 1–3)	ID or IN with 10^5^ TCID_50_ JEV Nakayama strain (GIII)	Nasal shedding, tissue dissemination pattern, histologic lesions, and immune responses were similar between the pigs infected via ID or IN route JEV replication in the brain of pigs is mostly efficiently suppressed, predominantly by type I interferon-independent activation of OAS1 expression and increased interferon-gamma activity	[66]
3-week-old white-line crossbreed piglets (groups of *n* = 2–5)	ID with 10^7^ TCID_50_ JE-91 strain (GI-b) with or without the addition of mosquito SGE	In contrast to the enhancement in arboviral diseases caused by mosquito saliva reported in mouse models, SGE reduced the severity of diseases caused by JEV infection CNS tissue viral loads did not differ significantly, and no demonstrable effects on viremic titers were observed with the co-inoculation of SGE and JEV	[51]

IV = intravenous; ID = intradermal; IN = intranasal; SQ = subcutaneous; JEV = Japanese encephalitis virus; WNV = West Nile virus; G = genotype; DPI = days post-infection; SPF = specific pathogen free; OAS1 = 2′-5′-oligoadenylate synthetase 1; CNS = central nervous system; SGE = salivary gland extract.

**Table 2:** List of important JEV research questions.

Examples of Knowledge Gaps in JEV Transmission
Can JEV be maintained in reservoir or amplifying hosts via vector-free transmission? What significant role, if any, does vector-free transmission play in the evolution of JEV? How is JEV maintained in temperate regions of Asia, where competent mosquito species are not present year-round? In what specific ecologic condition(s), if any, is vector-free transmission of JEV important? What role does persistent JEV infection of pigs play in viral maintenance or transmission?

JEV = Japanese encephalitis virus.

## Data Availability

Not applicable.
